# Epidemiology Characteristics of Constipation for General Population, Pediatric Population, and Elderly Population in China

**DOI:** 10.1155/2014/532734

**Published:** 2014-10-16

**Authors:** Huikuan Chu, Likun Zhong, Hai Li, Xiujing Zhang, Jingzhi Zhang, Xiaohua Hou

**Affiliations:** Division of Gastroenterology, Union Hospital, Tongji Medical College, Huazhong University of Science and Technology, 1277 Jiefang Road, Wuhan 430022, China

## Abstract

*Objective*. To acquire more data about the epidemiologic characteristics of constipation in different kinds of populations in China. *Methods*. Using “constipation” and “China” as search terms; relevant papers were searched from January 1995 to April 2014. Data on prevalence, gender, diagnostic criteria, geographical area, educational class, age, race, and physician visit results were extracted and analyzed. *Results*. 36 trials were included. Prevalence rates of constipation in elderly population (18.1%) and pediatric population (18.8%) were significantly higher than that in general population (8.2%). Prevalence of constipation defined by non-Rome criteria was higher than that by Rome criteria in general population. Prevalence rates of constipation were different for different geographical area. People with less education were predisposed to constipation. In pediatric population, prevalence of constipation was the lowest in children aged 2–6 years. Prevalence of constipation in ethnic minorities was higher than that in Han people. People with constipation were predisposed to FD, haemorrhoid, and GERD. Only 22.2% patients seek medical advice in general population. *Conclusions*. In China, prevalence of constipation was lower compared with most of other countries. The factors including female gender, diagnostic criteria, geographical area, age, educational class, and race seemed to have major effects on prevalence of constipation.

## 1. Introduction

Constipation is a common problem in the clinical practice. In Western countries, prevalence rate of constipation was reported to be between 2% and 28% [1–4] and was more common among women [5]. In Hong Kong, China, 14% of the general population had this complaint [6]. In recent years, the prevalence of constipation is increasing in China. Although a range of studies concerning epidemiology of constipation has been carried out, results were not uniform. It was reported by Shi et al. [7] and Xiong et al. [8] that prevalence of constipation in rural area was significantly higher compared with that in urban area, while Kan et al. [9] and Zhao et al. [10] reported that there was no significant difference in the prevalence between rural and urban area. On the other hand, Zhao et al. [10] and Wei et al. [11] indicated that there was no association between constipation and education, but Xiong et al. [8] reported that prevalence of constipation increased with less education. Moreover, no systematic review has been reported to evaluate the epidemiology of constipation in China. Therefore, the purpose of this systematic review was to acquire more data about the epidemiologic characteristics of constipation in China.

## 2. Materials and Methods

### 2.1. Search Strategy

Using constipation, functional constipation, chronic constipation, idiopathic constipation, prevalence, rate, proportion, epidemiology, morbidity, and China as search terms, relevant papers in English and non-English were searched in PubMed, Embase, Google scholar, ISI Web of Science, CJFD, WeiPu, and WanFang from January 1995 to April 2014. The reference lists of published articles were then used to locate other relevant studies, and the papers that fulfill the inclusion criteria were selected for further investigation. We also wrote email to corresponding authors of the relevant articles we found and asked whether the authors had knowledge of other relevant articles not yet published. When an article provided insufficient information to enter data for an analysis, we wrote email to corresponding author and asked for the needed information.

### 2.2. Inclusion Criteria and Exclusion Criteria

Inclusion criteria were as follows: (i) studies about prevalence of constipation in China were considered; (ii) results published in full text form; (iii) the data including at least two aspects among prevalence, sex, diagnostic criteria of constipation, age, educational class, race, and physician visit; (iv) diagnosis of constipation defined according to a questionnaire or a physician's diagnosis or based on the self-reported, Rome II or III criteria.

Exclusion criteria were as follows: (i) repetitive studies or patients researched that were duplicated; (ii) abstracts; (iii) studies with insufficient data; and (iv) studies that only investigated the special population, such as students, soldiers, and women.

### 2.3. Study Selection

All papers were examined separately by two reviewers (Huikuan Chu and Likun Zhong). If there was disagreement, all inconsistencies on article selection were resolved by discussion. If the abstracts met the first two inclusion criteria, the full texts were found manually by contacting the author or other methods to make sure of the integrity and reliability of the data. Choose a recent study with sufficient information if there were several studies written by the authors with the duplicate patients. Otherwise, choose all the papers if the patients were not duplicate in the papers written by the same author.

### 2.4. Data Extraction

Data were extracted from each study mainly focused on prevalence, gender, diagnostic criteria, geographical area, educational class, age, race, and physician visit results.

### 2.5. Quality Evaluation

Quality of each study was assessed through the guidelines developed by Loney and colleagues for critical appraisal of research on prevalence and incidence. The scoring system is based on 8 items including setting with random sample or whole population, unbiased sampling frame, adequate sample size, standard criteria used for measurement, health outcome measured in an unbiased fashion, adequate response rate, reports prevalence or incidence with conidence interval by subgroup and detailed description of study subjects. A score of 0 or 1 is assigned for each item, for a possible range of 0 to 8 [12].

### 2.6. Statistical Analysis

Data were analyzed through SPSS Version 17.0 (SPSS Inc., Chicago, IL, USA) and Excel. Proportions were evaluated by standard formula and proportions of various groups were compared by *χ* ^2^ test.

## 3. Results

### 3.1. Study Descriptions

A total of 315 potentially relevant studies were identified using the search strategy. After the first round of analysis, 247 of them were excluded and 32 studies were excluded from further evaluation (duplicate 7, abstract 5, gastrointestinal outpatient 6, university students 7, soldiers 3, women 2, nurse 1, and coal miners 1). Finally, 36 relevant studies (29 in Chinese and 7 in English [6, 10, 13–17]) were included in the meta-analysis for data extraction: 15 in the general population, 14 in pediatric population, and 7 in elderly population (Tables 1, 2, and 3).

#### 3.1.1. Prevalence of Constipation

The overall prevalence rates of constipation in 36 different population groups widely ranged from a low 3.1% in a pediatric population in Guangdong Province [18] to a high 67.8% in an elderly population in Tangshan City [19]. More than half of the studies with prevalence rates range from 3% to 10%. A total of 69796 cases in general population were studied and 5732 patients with constipation were found, with a prevalence rate of 8.2%. 2699 patients were found in 14893 cases in elderly population and the prevalence rate was 18.1%. The mean value of prevalence rate in pediatric population was 18.8% (16943/90091). There were significant differences among general population, pediatric population, and elderly population (*χ* ^2^ = 51956.5, *P* < 0.001).

#### 3.1.2. Diagnostic Criteria Differences in the Prevalence of Constipation

As we know, the diagnostic criteria of constipation range from self-reported to consensus criteria like Rome II criteria and Rome III criteria [20, 21]. The diagnosis of constipation could be on the basis of symptoms self-reported by the individual or defined according to a questionnaire, according to a physician's diagnosis, or based on the Rome II or Rome III criteria. We defined the diagnostic criteria that did not conform to the Rome criteria as non-Rome criteria. Considering the limited number of studies for each criteria, we just investigated the difference between non-Rome criteria and Rome criteria which included Rome II criteria and Rome III criteria.

In general population, 12 papers that defined constipation by Rome criteria were included and the average prevalence of constipation was 6.9%, which was lower than that by non-Rome criteria reported by 3 papers with a prevalence rate of 13.4% (*χ* ^2^ = 24635.1, *P* < 0.001). As one paper reported the prevalence of constipation defined by Rome criteria, differences between Rome criteria and non-Rome criteria were not assessed in elderly population. As we know, prevalence of constipation was different for pediatric population with different age and prevalence rate in Hong Kong being higher than that in Chinese Mainland; we only investigated the difference of prevalence rate for children aged 2–14 years in Chinese Mainland. 2 papers defined constipation by Rome criteria and 3 papers with non-Rome criteria. The result showed that prevalence of constipation was 4.7% by Rome criteria and 4.3% by non-Rome criteria. There was no significant difference between two criteria (*χ* ^2^ = 0.6, *P* = 0.43).

During the following comparing, we just compared the cases diagnosed by Rome criteria in general population and the cases based on same diagnostic criteria in elderly population considering the diagnostic criteria differences in the prevalence of constipation.

#### 3.1.3. Gender Differences in the Prevalence of Constipation

There were 27 trails written about the gender of patients: 11 in general population, 4 in elderly population, and 12 in pediatric population. The result of our research implied that prevalence of constipation in female was higher than that in male for all groups, with the ratio (male/female) of 1 : 1.4 in general population, 1 : 1.1 in elderly population, and 1 : 1.1 in pediatric population (Table 4). Generally speaking, prevalence of constipation in women was higher and females were more likely to report FC than males.

#### 3.1.4. Age Differences in the Prevalence of Constipation

**Figure 1:**
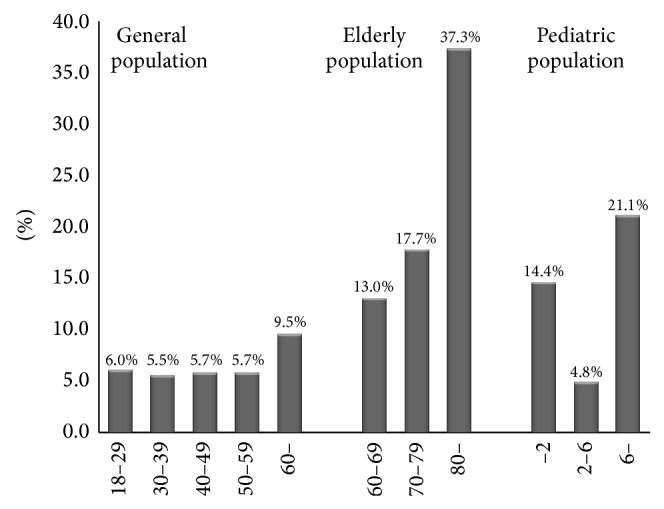
Age difference in the prevalence of constipation. Prevalence of constipation was the lowest in people aged 30–39 years old in general population and prevalence of constipation increased with age for people older than 30 in general population and elderly population. Children aged 2–6 years had the lowest prevalence of constipation and older than 6 years had the highest prevalence in pediatric population.

In general population, prevalence of constipation was the lowest in people aged 30–39 years old and prevalence of constipation increases with age for people older than 30 (in general population *χ* ^2^ = 357.1, *P* < 0.001). In elderly population, the results indicated that prevalence of constipation increased with age. There were significant differences among different aged people in elderly population (*χ* ^2^ = 3080.8, *P* < 0.001). In pediatric population, children aged 2–6 years had the lowest prevalence of constipation (4.8%) and children older than 6 years had the highest prevalence rate (21.1%). There were significant differences among different aged people in pediatric population (*χ* ^2^ = 1416.3, *P* < 0.001) (Figure 1).

#### 3.1.5. Regional Differences in the Prevalence of Constipation

**Figure 2:**
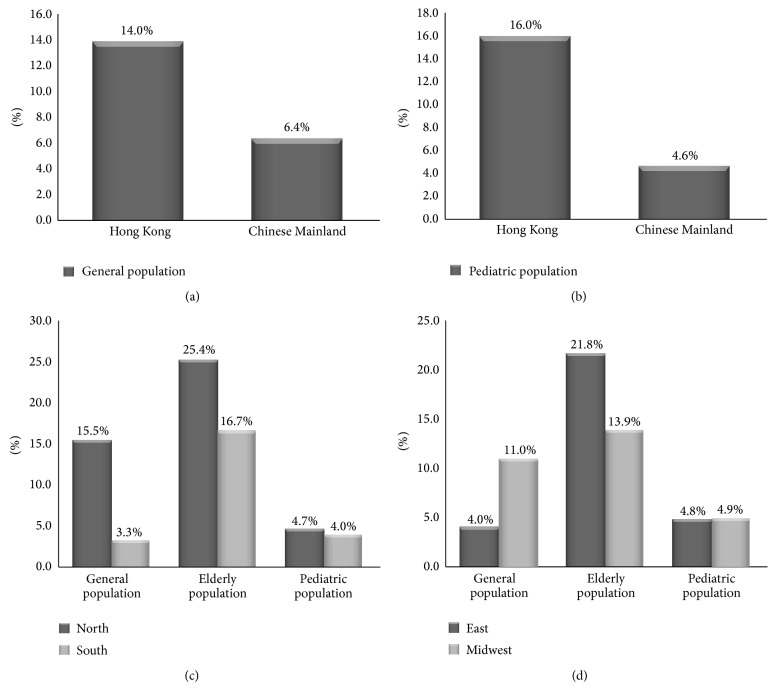
Regional difference in the prevalence of constipation. (a) In general population, the prevalence rate of constipation in Hong Kong was higher than that in Chinese Mainland. (b) In pediatric population, the prevalence rate of constipation in Hong Kong was higher than that in Chinese Mainland. (c) Prevalence rate of constipation in Northern area was significantly higher than that in Southern area in general population and elderly population, while there was no significant difference between Northern area and Southern area in pediatric population. (d) Prevalence rate of constipation in Eastern area was significantly lower than that in Midwestern area in general population, while prevalence rate in Eastern area was significantly higher than that in Midwestern area in elderly population, and there was no significant difference between Eastern area and Midwestern area in pediatric population.

In general population, we just compared the cases diagnosed by Rome II criteria considering the diagnostic criteria differences in the prevalence of constipation. The prevalence rate of constipation in Hong Kong was significantly higher than that in Chinese Mainland (14.0% versus 6.4%; *χ* ^2^ = 43277.5, *P* < 0.001). Prevalence rate was 15.5% in Northern area and 3.3% in Southern area and there were significant differences between them (*χ* ^2^ = 7403.5, *P* < 0.001). Prevalence rate in Eastern area was 4.0% and that in Midwestern area was 11.0%. There was significant difference between them (*χ* ^2^ = 872.7, *P* < 0.001). In elderly population, as there were limited studies that reported the epidemiology of Hong Kong, we only analyzed the differences between Northern and Southern area and between Eastern and Midwestern area. Prevalence rates were 25.4% in Northern area, 16.7% in Southern area, 21.8% in Eastern area, and 13.9% in Midwestern area. The result implied that there were significant differences for both of them (between Northern area and Southern area *χ* ^2^ = 117.1, *P* < 0.001 and between Eastern area and Midwestern area *χ* ^2^ = 5512.2, *P* < 0.001). In 2- to 15-year-old children, the prevalence of constipation in Hong Kong was 16.0% and that in Chinese Mainland was 4.6%. There were significant differences between them (*χ* ^2^ = 711.2, *P* < 0.001). The prevalence rates were 4.7% in Northern area, 4.0% in Southern area, 4.8% in Eastern area, and 4.9% in Midwestern area. There were no significant differences between Northern and Southern area (*χ* ^2^ = 2.1, *P* = 0.15), as well as between Eastern and Midwestern area (*χ* ^2^ = 0.15, *P* = 0.7) (Figure 2).

#### 3.1.6. Urban and Rural Differences in the Prevalence of Constipation

4 trails in general population and 3 papers in elderly population investigated differences of the prevalence of constipation between urban and rural area. In general population, prevalence of constipation in urban area was 6.7%, which was significantly lower than that in rural area with a prevalence rate of 7.2% (*χ* ^2^ = 154.4, *P* < 0.001). In elderly population, prevalence rate in urban area was 12.0%, lower than that in rural area with a prevalence rate of 14.1% (*χ* ^2^ = 10.5, *P* < 0.001). Four papers assessed the difference of the prevalence of constipation between urban and rural area people in pediatric population. Prevalence of constipation in urban area (4.1%) was lower than that in rural area (4.6%); however, there was no significant difference between them (*χ* ^2^ = 0.45, *P* = 0.5).

#### 3.1.7. Educational Differences in the Prevalence of Constipation

**Figure 3:**
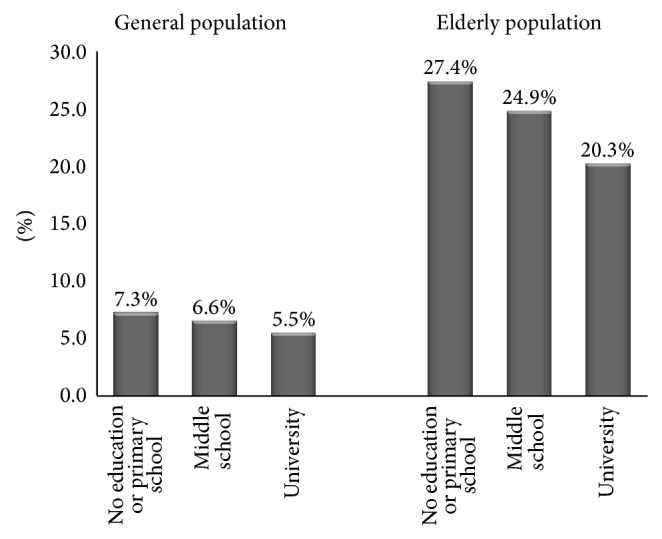
Education difference in the prevalence of constipation. Prevalence rate of constipation increased with less education in general population and elderly population.

In general population and elderly population, prevalence rate of constipation increased with less education (in general population *χ* ^2^ = 8468.4, *P* < 0.001; in elderly population *χ* ^2^ = 2618.7, *P* < 0.001) (Figure 3).

#### 3.1.8. Racial Differences in the Prevalence of Constipation

In general population, 2 papers reported the race difference in the prevalence of constipation. 149 patients were found in 811 cases in ethnic minorities, with a result that prevalence of constipation was 18.4%, which was higher than that in Han people with a prevalence rate of 12.5% (*χ* ^2^ = 22.2, *P* < 0.001).

#### 3.1.9. Incidence of Other Digestive Disorders for People with Constipation

In general population, 2 papers reported the relationship between constipation and FD, with a result of people with constipation being predisposed to FD (*χ* ^2^ = 712.0, *P* < 0.001). Kan et al. [9] reported that prevalence of hemorrhoid (*χ* ^2^ = 312.2, *P* < 0.001) and GERD (*χ* ^2^ = 60.3, *P* < 0.001) was higher in patients with constipation.

#### 3.1.10. Physician Visit

We could only assess the physician visit of general population and pediatric population because of the limited report of elderly population. 6 trails studied the physician visit of general population with a result of 22.2%; 5 papers reported the physician visit of pediatric population with a result of 27.8%. On the other hand, the symptoms improved in 50.2% patients with constipation by increasing activity or more fiber intake instead of visiting physician in general population. 17.5% children improved their constipation by more activity or more fiber intake.

## 4. Discussion

The prevalence rate of constipation in general population in China was 8.2% in this review, which was lower than most of other countries such as 17.1% in Europe and Oceania [22], 16.5% in Korea [23], 19.4% in USA [24], and 14.9%–27.2% in Canada [25]. The difference may partially be due to the different dietary habits [26, 27]. Constipation in individuals with previously normal bowel function was associated with specific dietary patterns, such as low fiber intake which is involved with the pathogenesis of constipation [15, 28, 29]. Most people in our country have dietary habits consisting of increased consumption of fibers. On the other hand, life style and physical inactivity [30, 31] were risk factors for constipation. Compared with other countries, people in our country have different life style and more physical activity [32].

In general population, prevalence rate of constipation defined with Rome criteria was lower than that with non-Rome criteria. This may be due to different diagnostic criteria resulting in significant differences in prevalence rates [33] and every symptom was explicitly defined in Rome criteria whereas symptoms were simply described in non-Rome criteria, resulting in some patients who were included by non-Rome criteria rather than excluded by Rome criteria.

We found that females were more likely to report FC than males, which was consistent with that in North America [1, 34], Europe, and Oceania [22]. This predominance of females may be attributed to hormonal factors, inducing a higher risk of constipation during the luteal phase of the menstrual cycle, under the effects of progesterone, anatomic features of female pelvic floor, and the pelvic floor muscles and nerves damaged during delivery [35–38]. Posterior colpocele was an independent risk factor for constipation [39].

The prevalence of constipation increased with age in general population for people older than 30 and in elderly population. The trend that prevalence of constipation increased with age was consistent with other countries [22, 34], which may be due to the healthy status, medication [40], and physical inactivity [30]. For people older than 80 years old, the prevalence rate reached to 37.7%, which was in the range of the elderly constipation epidemiology in Western countries [1, 41–48]. The underlying reason for constipation in elderly population include insufficient intake of fluid and dietary fiber, reduced daily activities, lack of exercise, and diseases such as neurological and metabolic disorders [44–46, 49], in addition to drugs [50–57]. Healthy condition becomes poor and incidence of diabetes [58, 59], stroke, Parkinson's disease, and local neurogenic disorders [60–62] increased with age and people have to take more medicine to treat their disease condition. On the other hand, physical activities would be reduced with age. We should pay attention to that the established fact that the higher prevalence of constipation for elderly can cause a range of complications, such as anorectal pathology (e.g., fissures, hemorrhoids, rectal prolapse, etc.), sigmoid volvulus, fecal impaction, fecal incontinence, and urinary dysfunction [21, 63, 64].

In pediatric population, prevalence of constipation was highest in children older than 6 years who were school-aged children, which may be attributed to the high level of psychological stress caused by fast-paced and greatly competitive school environment [16].

In general and elderly population, prevalence of constipation in Northern area was higher than that in Southern area, which might be due to different dietary habits and socioeconomic status [65]. It was reported that increased intakes of rice at breakfast, lunch, and dinner were all associated with a decreased prevalence of constipation and low socioeconomic status is a risk factor for constipation [66]. In China, people in Southern area have the dietary habits with rice and socioeconomic status in Southern area was higher than Northern area. On the other hand, we could not exclude the cold and dry weather of Northern area which result in a higher prevalence of constipation [65]. Prevalence of constipation in Midwestern area was higher than that in Eastern area in general population and this may be attributed to the low socioeconomic status and dry weather of Midwestern area [7].

In general population and elderly population, prevalence of constipation in rural was higher than that in city. This may be attributed to the low socioeconomic condition [66] and less educational class [67]. In China, socioeconomic condition and educational class was lower for people living in rural area.

In this study, we found that there were racial differences in the prevalence of constipation. Prevalence of constipation in ethnic minorities was higher than that in Han people. This was similar with the result of America that prevalence of constipation was higher in non-Caucasians than in Caucasians [1]. This might be caused by the different dietary habits [26] and living style between different races.

In conclusion, compared with most of other countries, prevalence rate of constipation in China was lower and more common for women. The prevalence of constipation in general population was influenced by diagnostic criteria, area, age, educational class, and race. Risk factors of constipation in elderly population include geographical area, rural area, age, and educational class and that in children was mainly due to age. Only a small proportion of patients suffering from constipation seek medical consultation.

## Figures and Tables

**Table 1:** Characteristics of included papers (in general population).

Author	Area of origin	Population	Year of publication	Diagnosed criteria	Number of investigated people	Number of patients	Quality score
Zhao et al. [10]	Five cities in China	18–80 years	2011	Rome II	16078	948	7
Cheng et al. [6]	Hong Kong	18–80 years	2003	Rome II	3282	458	7
Wei et al. [11]	Guangzhou City	>18 years	2001	Rome II	2892	108	5
Xiong et al. [8]	Guangdong Province	18–80 years	2004	Rome II	3931	159	6
Shen et al. [68]	Shanghai City	18–97 years	2012	Rome III	7648	211	4
Kan et al. [9]	Tianjin City	>18 years	2003	<3 BMs/week	7220	838	5
Tang et al. [69]	Anhui Province	>18 years	2008	Rome III	3709	139	4
Zuo and Han [70]	Henan Province	2–92 years	2012	Rome III	6102	1108	4
Shi et al. [7]	Xinjiang Autonomous Region	>10 years	2010	Rome II	1354	285	4
Lv et al. [71]	Nanchang City	10–91 years	2005	Rome II	3745	122	4
Guo et al. [72]	Beijing City	18–70 years	2001	Rome II	2486	151	6
Xiang et al. [73]	Chongqing City	>18 years	2004	Rome II	1492	47	4
Zhang et al. [74]	Sichuan Province	11–90 years	2008	Rome II	2912	96	5
Zhang et al. [75]	Henan Province	General population	2008	<3 BMs/week	1838	163	4
Liu et al. [76]	Hangzhou City	1 y–99 years	2004	<2 BMs/week	5107	899	4

**Table 2:** Characteristics of included papers (in pediatric population).

Author	Area of origin	Population	Year of publication	Diagnosed criteria	Number of investigated people	Number of patients	Quality score
Xu et al. [18]	Guangdong Province	2–6 years	2008	Rome III	1022	32	4
Zhou et al. [77]	Guangdong Province	6-18 years	2010	Rome III	2075	130	5
Zhou et al. [78]	Shanghai City	10–18 years	2008	Rome II	51956	13467	5
Wang et al. [79]	Beijing City	2–14 years	2003	<3 BMs/week or hard	1001	38	5
Liu et al. [80]	Chengdu City	2–7 years	2007	Strain or hard or <3 BMs/week	1132	54	3
Hu [81]	Xian City	2–12 years	2003	Strain or hard or <3 BMs/week	1001	44	4
Zhang et al. [82]	Five cities in north	4–14 years	2010	Rome III	19286	913	4
M. F. Chan and Y. L. Chan [13]	Hong Kong	3–5 years	2010	<3 BMs/week	383	28	6
Lee et al. [14]	Hong Kong	3–5 years	2008	Rome II	368	106	6
Ip et al. [15]	Hong Kong	3–5 years	2005	Rome II	561	166	5
Liang and Song [83]	Guangdong Province	6–8 months	2003	Strain or hard or <3 BMs/week	287	78	3
Liu et al. [84]	Shanghai City	0–24 months	2009	Rome III	5030	690	4
Tam et al. [17]	Hong Kong	6–15 years	2012	Rome III	2318	282	5
Zhou et al. [16]	Shanghai City	12–18 years	2011	Rome III	3671	915	6

**Table 3:** Characteristics of included papers (in elderly population).

Author	Area of origin	Population	Year of publication	Diagnosed criteria	Number of investigated people	Number of patients	Quality score
Li et al. [85]	Beijing City	>60 years	2000	Strain or hard or <3 BMs/week	1434	291	4
Cai et al. [86]	Nanjing City	>60 years	2004	Strain or hard or <3 BMs/week	3731	897	3
Yu et al. [65]	Beijing City	>60 years	2001	Strain or hard or <3 BMs/week	8252	950	4
Pan and Shen [87]	Beijing City	>60 years	2008	Rome II	114	45	4
Liu et al. [19]	Tangshan City	>60 years	2007	Strain or hard or <3 BMs/week	545	369	3
Guo et al. [88]	Tianjin City	>70 years	2004	Strain or hard or <3 BMs/week	76	32	3
Huang and Zhang [89]	Sichuan Province	>60 years	1999	<3 BMs/week	741	115	4

**Table 4:** Gender differences in the prevalence of constipation.

	Male	Female	Ratio male/female
General population	5.6%	8.1%	1 : 1.4
Elderly population	14.9%	16.7%	1 : 1.1
Pediatric population	19.1%	21.6%	1 : 1.1
